# Campus Sexual Assault: A Qualitative Review and Meta-Synthesis of Students’ Experiences of Campus Prevention Initiatives

**DOI:** 10.1177/15248380241297332

**Published:** 2024-11-25

**Authors:** Karen McQueen, Jodie Murphy-Oikonen, Jasmin Hamm

**Affiliations:** 1 Lakehead University School of Nursing, Thunder Bay, ON, Canada; 2 Lakehead University School of Social Work, Thunder Bay, ON, Canada

**Keywords:** campus sexual assault, intervention, prevention

## Abstract

Sexual violence is a worldwide issue that impacts many individuals, often with serious and long-lasting effects. Students represent a high-risk group for sexual violence on campuses. As a result, various government initiatives have called for Universities and Colleges to develop policies and sexual violence prevention initiatives. However, much of the focus has been on the effect of the initiatives with less attention to students’ experiences. Thus, the purpose of this qualitative systematic review with meta-synthesis was to critically appraise and synthesize the evidence (e.g., themes) regarding students’ experiences with campus sexual assault prevention initiatives. The Preferred Reporting Items for Systematic Reviews and Meta-Analysis and Joanna Briggs Institute guidelines were followed. Six databases were systematically searched, which yielded 2,090 papers. This resulted in 21 published papers meeting the inclusion criteria of a primary, English language, qualitative or mixed-methods study exploring students’ experiences of campus sexual assault initiatives. Two researchers independently extracted data and completed quality appraisals. The meta-synthesis identified four synthesized findings: (1) dichotomous perceptions of sexual violence initiatives, (2) the need for enhanced awareness, (3) modality matters, and (4) intended and unintended outcomes. Overall, these findings suggest that students valued the attention to the issue of sexual violence; however, they identified concerns that warrant attention. This has important implications for program and policy development as having programs that meet students’ needs may result in enhanced student engagement which may, in turn, lead to increased efficacy. Moreover, initiatives that may result in harm to survivors require careful consideration.

## Background

Sexual violence (SV) is a serious problem on University and College campuses (Cantor et al., 2015). A systematic review that included 34 studies published between 2000 and 2015 found that approximately 20% of women and 6% of men had experienced sexual assault during College (Fedina et al., 2018). This is consistent with SV statistics from Universities in the United States (Cantor et al., 2015) and Canada (SARE Study Team et al., 2014). However, many speculate that the prevalence is much higher as sexual assault is one of the most underreported crimes (Benoit et al., 2015). Furthermore, populations who may be at higher risk of sexual victimization have not been captured in much of the campus sexual assault (CSA) literature (Fedina et al., 2018). Significantly higher rates of sexual assault have been found among lesbian, gay, bisexual, queer (Eisenberg et al., 2021), and trans students (Hoxmeier & Madlem, 2018) than those who identify as heterosexual. Other vulnerable groups that are under-represented include culturally and racially diverse populations such as Indigenous women (Du Mont et al., 2017), women with a disability (Basile et al., 2016), and those with a history of victimization (Carey et al., 2015; Fisher et al., 2010).

The negative outcomes of SV for survivors are well documented (Dworkin et al., 2017) and include a host of physical, mental, social, and sexual sequelae that can leave the well-being of survivors significantly compromised (Bordere, 2017; Wong & Balemba, 2016). Students who have experienced sexual assault are also more likely to engage in risky behaviors such as binge drinking and drug use (Blayney et al., 2019), have lower academic success (Molstad et al., 2023), and poorer mental health (Carey et al., 2018).

Several factors place students at increased risk of sexual assault at the individual and organizational levels. These include a confluence of factors such as increased alcohol consumption, multiple sexual partners, peer pressure, young adulthood, and limited supervision (Lichty et al., 2008), as well as the availability and accessibility of sexual assault services and resources, alcohol policies, and athlete and fraternity cultures that minimize claims of sexual assault (Moylan & Javorka, 2020). Many have called on the need for prevention initiatives on campus to mitigate risk to students and decrease negative outcomes associated with sexual assault. Government regulations (e.g., US Campus SaVE Act; Title IX; Bill 132 [Ontario Government]) over the past decade have led many institutes of higher learning (IHL) to implement prevention and reporting policies (Government of Ontario, 2015; Reynolds, 2019; US Congress, 2013). As such, there has been an increase in the development and implementation of SV prevention programs and resources for students on campus (Carey et al., 2018).

The growing body of evidence on CSA preventative interventions includes sexual assault education programs (Anderson & Whiston, 2005; Heard et al., 2024), bystander education (Jouriles et al., 2018; Katz & Moore, 2013), male-targeted programs (Hammock et al., 2022; Wright et al., 2020), peer education theater (Christensen, 2014; McMahon et al., 2014), among others. In addition, secondary and tertiary prevention efforts (e.g., counseling, advocacy resources, reporting policies) to reduce the impact of SV have also been increasing on campuses (Holland et al., 2021). The quantitative literature, which includes systematic reviews of prevention interventions, has demonstrated beneficial effects on some program outcomes such as improvements in bystander efficacy, intentions to help others (Jouriles et al., 2018; Katz & Moore, 2013), attitudes regarding rape, empathy, and knowledge (Anderson & Whiston, 2005; Kettrey et al., 2023). However, less is clear about whether the interventions are effective in preventing or decreasing sexual assaults (Katz & Moore, 2013; Kettrey et al., 2023; Wright et al., 2020). In addition, limitations have been identified regarding the evaluation of sexual assault interventions. In particular, much of the research has been conducted primarily among white, female students in the early years of post-secondary education (Fedina et al., 2018). Furthermore, while many quantitative studies provide evidence regarding the effectiveness of programs, less attention has been given to the perspective of students who have participated in the programs and whether they are appropriate and acceptable. Students have explicitly stated that they want to be meaningfully consulted in gender-based violence policies, prevention, and support initiatives (Protech & Rosser, 2021). As such, there is a need to take into account evidence from qualitative studies to better understand students’ experiences regarding CSA prevention initiatives and whether such initiatives apply to their sense of safety or the safety of others.

Existing qualitative studies have explored various CSA prevention initiatives. For example, Worthen and Wallace (2021) critically examined sexual assault survivors’ responses to mandatory online CSA education programs. The researchers described survivors’ experiences of being triggered and/or retraumatized, and the need to consider survivors’ experiences in mandatory training. Additional examples of student experiences include the perception of CSA programming from female students of color (Karunaratne & Harris, 2022), exploring the experiences of students with theater-based, peer-led sexual assault prevention (Christensen, 2014), and gaining insight into college sexual assault survivor’s perceptions of university mandatory reporting guidelines (Holland et al., 2021). These studies provide context regarding how interventions may or may not be meeting the needs of students (Hubach et al., 2019); however, the findings are diverse. Christensen (2014) found that students were generally positive regarding theater-based peer education and felt it positively impacted their attitudes and behaviors. Whereas the other studies identified barriers such as a lack of engagement by students in large group education sessions, the perpetuation of rape myths (Karunaratne & Harris, 2022), and the potential for harm with some university policies (e.g., mandatory reporting; Holland et al, 2021). While these individual studies provide rich descriptions of students’ experiences, a qualitative synthesis of the findings would better inform evidence-based practice and decision-making (Lockwood et al., 2015). In addition, a synthesis of students’ experiences would recognize and validate students’ and survivors’ experiences as a form of expert knowledge (Protech & Rosser, 2021). Therefore, the purpose of this qualitative systematic review with meta-synthesis was to identify, critically appraise and synthesize the evidence (e.g., themes) of all relevant studies that used qualitative methodologies on students’ experiences with CSA prevention initiatives.

## Methods

We followed the Joanna Briggs Institute (JBI) methodology for conducting qualitative system reviews and the Preferred Reporting Items for Systematic Reviews and Meta-Analysis (PRISMA) guidelines (Page et al., 2021). For this review, CSA prevention initiatives were broadly defined to include any sexual assault/SV initiative (e.g., education, policies, resources) provided to students from institutions of higher learning. We defined students as those currently enrolled in post-secondary education (college or university) and/or a recent graduate (alumnus). Lastly, students’ experiences and perceptions were defined to include students’ attitudes, experiences, and perceptions.

### Data Sources and Search Strategy

We utilized a three-step search strategy to find published studies. An initial limited search was undertaken by the primary researcher to find articles on the topic of campus-based SV initiatives and to identify keywords and index terms. A second search using all identified keywords and index terms was undertaken in six databases (PsychInfo, Social Work Abstracts, EBSCO, Proquest, CINAHL, and PubMed) with a university librarian experienced in systematic review searches. Third, for all included studies, we performed a hand search of the reference list and a prospective search in Web of Science for additional articles to ensure articles were not missed. The final search for this systematic review was conducted on February 27, 2024. The full search strategy from the library scientist for PubMed is included in Supplemental Table 1.

### Eligibility Criteria

As the focus of the review was on students’ experiences of CSA initiatives, we included (1) all studies (qualitative or mixed methods) with qualitative data (themes) on the experiences or perceptions of students regarding SV initiatives provided by colleges or universities and (2) peer-reviewed articles of primary studies published in English over the past 10 years. Ten years was selected to ensure that the CSA initiatives were current. Studies were excluded if (1) the participants included students and other groups (e.g., faculty/staff) where the student data were integrated, (2) studies that did not include a specific campus initiative (e.g., studies that were conducted with students but not linked to a specific initiative or school), and (3) studies that would be considered at a high risk of bias with a critical appraisal score lower than 4 out of 10.

### Data Management and Study Selection

All citations from the search were uploaded into Covidence software by the university librarian and duplicates were removed. Two members of the research team (KM and JM-O) independently screened the title, abstracts, and full-text articles for eligibility in the systematic review as per the inclusion/exclusion criteria. When reviewing the articles, we specifically looked at the population (e.g., students), methodology (qualitative or mixed methods), data analysis (themes, quotes), and area of interest (students’ experiences with CSA initiatives). We were careful not to disregard studies labeled as quantitative in case they had a qualitative component including open-ended questions. A re-review of articles was conducted if there were discrepancies to achieve consensus. Next, a research assistant reviewed all excluded articles to confirm exclusion decisions. No studies were identified for inclusion from the exclusion folder.

### Assessment of Methodological Quality

The included articles were independently assessed by two reviewers (JM-O, JH) using the JBI Quality Assessment and Review Instrument (JBI-QARI) (Joanna Briggs Institute, 2014). The critical appraisal checklist contains 10 items, yielding a total score of 10 points, with higher scores indicating a lower risk of bias. The checklist for qualitative research includes questions to assess the possibility of bias in the study design, conduct, and analysis (Lockwood et al., 2015). Any conflicts between the reviewers on the quality appraisal assessment were resolved with a third reviewer (KM). One article was removed from the review due to a high risk of bias with a low critical appraisal score (3/10) (Jozkowski & Sanders, 2012).

### Data Extraction

Qualitative data from full-text articles were extracted independently by two reviewers (KM and JH) using a modified version of the JBI template for data extraction for qualitative evidence (Joanna Briggs Institute, 2014). This included specific details about the study purpose, setting, population, sample, college/university sexual assault prevention program, and research methodology (see Table 1). All themes/subthemes and corresponding quote(s) were extracted as findings. Thereafter, the level of congruency between the findings and supporting quotes was ranked by the level of evidence (unequivocal, plausible, or unsupported) (Lockwood et al., 2015). The term equivocal signifies that the finding was accompanied by a quote that directly substantiated the theme, equivocal findings are accompanied by a quote that can be logically inferred from the data and unsupported indicates that the finding was not supported by the data (Lockwood et al., 2015).

**Table 1.** Characteristics of Included Studies.

Author/date	Country/setting	Purpose of study	University/college program services	Sample	Methodology	Data analysis
Acquaviva et al. (2023)	One US midwestern public university	Examine college students’ and alumni’ perceptions of online sexual assault training	Online sexual assault training (consent and respect)	*N* = 34; undergrad (*n* = 12), grad (*n* = 11), and alumni (*n* = 11)	Qualitative; semi-structured, face-to-face interviews	Systematic coding in two phases
Bloom et al. (2022)	Three public universities in California, USA	Assess students’ perceptions of campus sexual violence and sexual harassment (SVSH) resources, gaps in services, and recommendations for solutions for SVSH	Counseling and psychological services, campus advocacy resource and education offices, student health services, campus police department, and Title IX offices	*N* = 222 undergraduate students	Qualitative; community-based participatory research. Interviews (*n* = 86) and focus groups (*n* = 136)	Thematic analysis (Bruan and Clarke)
Christensen (2014)	One large state university in the US	Explores the experiences (i.e., beliefs, attitudes, opportunities) of students	Theater-based, peer-education, sexual assault prevention presentation	*N* = 56 undergraduate college students	Qualitative; focus groups	Line-by-line descriptive and analytic coding (Charmaz)
Chugani et al. (2021)	13 campuses across Western Pennsylvania and West Virginia, US	Understand perceptions of campus-based alcohol and sexual violence (SV) prevention programming	Campus-based alcohol and sexual violence (SV) prevention programming	*N* = 51 college students with disabilities with histories of sexual violence	Qualitative; semi-structured, face-to-face interviews	Thematic analysis (Bruan and Clarke)
Graham et al. (2021)	University in Aotearoa/New Zealand	To understand students’ decisions about attending (or not attending) sexual violence workshops	Bringing in the bystander (BITB) consent conversations	*N* = 28; groups of those who attended and did not attend	Qualitative; focus groups or individual interview	Thematic analysis (Bruan and Clarke)
Hammocket al. (2022)	A large public US north eastern institute of higher education	To understand the perceptions and experiences of sexual violence bystander prevention programs	1. Mandatory 20-minutes bystander training 2. Green dot training—voluntary 8-hour workshop from campus facilitators	*N* = 24 male participants; 3 groups were men of color and one group was Caucasian	Qualitative; focus groups	Thematic analysis (Braun and Clarke)
Heard et al. (2024)	One university in Australia	To evaluate an online sexual module	Online sexual violence and response education module	*N* = 13 undergraduate students	Qualitative; semi-structured interviews	Thematic analysis (Braun and Clarke)
Holland, Cipriano, and Huit (2021)	A large midwestern university campus in the US	To gain insight into college sexual assault survivors’ perceptions of university mandatory reporting	At the time of the study, the university used a selective mandatory reporting model	*N* = 40 undergraduate sexual assault survivors	Qualitative individual interviews	Thematic analysis (Braun and Clarke)
Hubach et al. (2019)	One large public university in south-central US	To determine students’ preferred method of sexual assault and sexual health information delivery	Mandatory online sexual assault prevention training	*N* = 24 university students; 2 groups of men and 2 women	Qualitative 4 focus groups based on gender	Thematic analysis (Corbin and Strauss)
Irvine-Collins, et al. (2023)	One large university in Australia	To investigate the impact of a marketing campaign implemented at the university	“Listen, support, refer”—a three-phase model	*N* = 11 students	Mixed method; qualitative portion 2 online focus groups	Thematic analysis (Braun and Clarke)
Karunaratne and Harris (2022)	Four higher education institutions in the Western US	To explore women of color student survivor perceptions of institutional CSA prevention	A Title IX Office; A counseling center; a sexual violence advocacy and education office	*N* = 44 women of color undergraduate students	Critical qualitative design; two semi-structured individual interviews	Thematic analysis (Braun & Clarke)
Lorenz et al. (2022)	Universities in the US	To explore the experiences of student/worker survivors with Title IX investigations	Most participants engaged with Title IX under Obama-era guidelines, some under Trump-era guidelines	*N* = 21 female undergraduate and graduate/worker survivors	Qualitative; grounded theory individual zoom interviews	Thematic analysis; grounded theory (Glaser and Strauss)
Machisa et al. (2023)	Female students in South Africa	Evaluate pilot feasibility of a sexual assault risk reduction program 1 year post-intervention	Ntombi Vimbela! (NV!), a sexual violence prevention intervention	*N* = 35 female students on eight campuses	Qualitative: in-depth phone interviews 1 year post-intervention	Thematic analysis (Braun and Clarke)
Mahlangu et al. (2022)	8 purposively selected campuses in South Africa	To evaluate the content of a sexual violence prevention intervention	Ntombi Vimbela! (NV!), a sexual violence prevention intervention	*N* = 118 female students first-year female students	Qualitative; pilot, participatory action research focus groups	Thematic analysis (Braun and Clarke)
Marques et al. (2020)	One mid-size university campus in Ontario, Canada	Explore students’ perceptions of sexual assault policies and services	University’s policies and procedures on sexual violence and harassment (not specified)	*N* = 15 female students	Mixed-methods design; in-depth individual interviews	Manual coding using simultaneous and thematic techniques (Saldana)
McMahon et al. (2021)	One large public university in the northeastern US	Explores the impact of a sexual violence program on student attitudes, self-perception, and behaviors	SCREAM theater a peer-led theater presentation on bystander education with two 6-minute skill-building sessions	*N* = 498 students	Mixed methods; qualitative open-ended questions on a survey	Inductive analysis, independent parallel coding, investigator triangulation
Philyaw-Kotov et al. (2023)	One large midwestern university in the US	To capture perspectives regarding desirable SA prevention program characteristics	Students complete various programs geared toward preventing SA	*N* = 35 undergraduate students; interview (*n* = 19) or focus group (*n* = 16)	Qualitative, pilot study	Double-coded transcripts for a priori and emerging themes
Sall and Littleton (2022)	One large Southeastern U.S. university	Examine college women rape survivors (attempted or complete rape) who disclosed to a campus resource	Title IX and/or police-mandated reporters, and confidential sources	*N* = 19 participants (college women survivors of an illegal SA)	Mixed methods: qualitative portion included 8 open-ended items	Narrative coding (Braun and Clarke)
Voth Schrag et al. (2024)	Five universities in a southwestern US state	To understand the approach campus-based advocacy (CBA) programs use to address safety and academic concerns	CBA programs typically focus on the safety and academic needs of survivors in college settings	*N* = 29 participants	Mixed methods: semi-structured interviews via Zoom, phone, or in-person	Thematic analysis (Braun and Clarke)
Worthen et al. (2021)	A large southern university in the US	To critically examine responses to a mandatory online sexual assault education program	Mandatory online sexual assault education program	*N* = 41 survivors or those who know survivors	Mixed methods: qualitative component included one open-ended question	Grounded theory approach (Charmaz)
Worthen et al. (2017)	One university located in a southern state of the US	To explore students’ reactions to an online sexual assault educational program	Campus-wide online mandatory sexual assault education program (45-slide PowerPoint)	*N* = 1,899 for the survey (did not specify how many replied to open-ended questions)	Mixed methods; qualitative component included an open-ended question	Charmaz’s approach to grounded theory

### Data Synthesis

We followed the JBI meta-aggregation approach to synthesize the findings (Lockwood et al., 2015). All qualitative extracted research study findings (e.g., themes and subthemes) that were extracted from the studies were pooled. This involved synthesizing the findings into categories based on similarity and meaning and then developing synthesized topics from the categories. Only findings that were evaluated as unequivocal or plausible were included in the data synthesis (Lockwood et al., 2015). Two reviewers (KM and JM-O) participated in the data synthesis.

## Results

**Figure 1.**
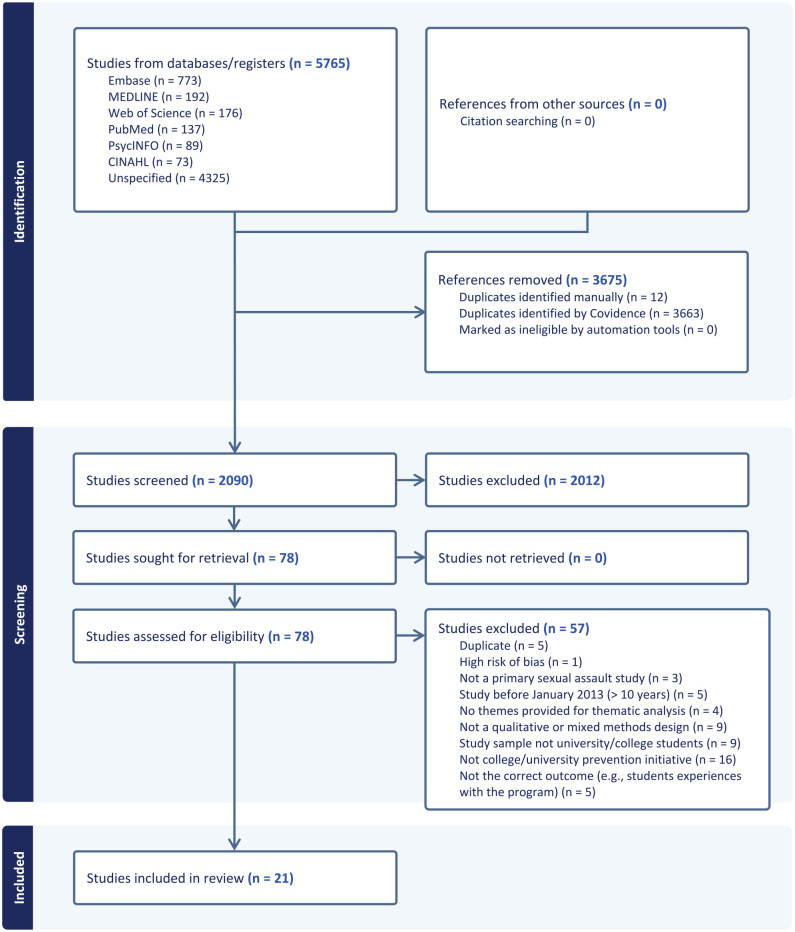
PRISMA flow diagram.

We identified a total of 5,765 citations from six databases. After the automatic removal of duplicates in Covidence (3,675), we reviewed the title and abstracts of 2,090 citations, and 78 articles were forwarded to full-text review. In all, 57 articles were removed as they did not meet the eligibility criteria. The most common reason for exclusion was the study did not evaluate a specific type of CSA initiative. Finally, 21 studies met the inclusion criteria and were included in the systematic review. See Figure 1 for the PRISMA flow diagram.

### Characteristics of Included Studies

The characteristics of the included studies are provided in Table 1. The studies were conducted in the United States (*n* = 15), Australia (*n* = 2), South Africa (*n* = 2), New Zealand (*n* = 1), and Canada (*n* = 1). All studies were qualitative (*n* = 14) or mixed-method designs (*n* = 7), which primarily used semi-structured interviews and/or focus groups for data collection. However, four of the mixed-methods studies reported their qualitative component as a written response to one or more open-ended questions. Various forms of thematic analysis were used to analyze data from the included studies.

The included studies explored diverse types of CSA programs including education initiatives (online, in-person, mandatory, theater-based, bystander), campus resources and policies, and reporting. The demographics of the student participants of the included studies varied. In many of the studies, there were more female participants than males (Acquaviva et al., 2023; Christensen, 2014; Chugani et al., 2021; Graham et al., 2021; Heard et al., 2024; Voth Schrag et al., 2024) and some studies had all female participants (Lorenz et al., 2022; Machisa et al., 2022; Mahlangu et al., 2022; Marques et al., 2020; Sall & Littleton, 2022). However, a few included studies had more diversity of participants regarding gender with a broader representation of men (Hammock et al., 2022; McMahon et al., 2021; Philyaw-Kotov et al., 2023), race/ethnicity (Bloom et al., 2022; Christensen, 2014; Hammock et al., 2022; Karunaratne & Harris, 2022; Marques et al., 2020; McMahon et al., 2021; Voth Schrag et al., 2024), and sexual orientation (Bloom et al., 2022; Christensen, 2014; Holland et al., 2021; Voth Schrag et al., 2024). Three mixed-methods studies only reported demographics on the total sample of participants and not those who participated in the qualitative component (Irvine-Collins et al., 2023; Worthen & Wallace, 2017, 2021).

### Quality Appraisal

The 21 included articles were assessed as having a moderate to low risk of bias (see Table 2). More than half (*n* = 12; 57.1%) of the articles had a low risk of bias on the quality appraisal checklist as the studies scored eight or more positive responses. The remaining studies (*n* = 9; 42.9%) were at moderate risk of bias with positive scores tallying six or seven. Two items consistently scored low with only eight studies (38.1%) including information about the authors’ philosophical perspective (question 1) and seven studies (33.3%) indicating the influence of the researcher on the research or vice-versa (question 7).

**Table 2.** Quality Appraisal of Included Studies.

Author/year	Q1	Q2	Q3	Q4	Q5	Q6	Q7	Q8	Q9	Q10	Total
Acquaviva et al. (2023)	N	Y	Y	Y	Y	U	N	Y	N	Y	**6**
Bloom et al. (2022)	Y	Y	Y	Y	Y	Y	Y	Y	Y	Y	**10**
Christensen (2014)	Y	Y	Y	Y	Y	Y	Y	U	Y	Y	**9**
Chugani et al. (2021)	U	Y	Y	Y	Y	Y	Y	Y	Y	Y	**9**
Graham et al. (2021)	U	Y	Y	Y	Y	Y	N	Y	Y	Y	**8**
Hammock et al. (2022)	N	Y	Y	Y	Y	N	N	Y	Y	Y	**7**
Heard et al. (2024)	Y	Y	Y	Y	Y	N	N	Y	Y	Y	**8**
Holland et al. (2021)	U	Y	Y	Y	Y	N	U	Y	Y	Y	**7**
Hubach et al. (2019)	Y	Y	Y	Y	Y	Y	Y	Y	Y	Y	**10**
Irvine-Collins et al. (2023)	U	Y	Y	Y	U	N	N	Y	Y	Y	**6**
Karunaratne and Harris (2022)	Y	Y	Y	Y	Y	Y	Y	Y	N	Y	**9**
Lorenz et al. (2022)	Y	Y	Y	Y	Y	Y	Y	Y	Y	Y	**10**
Machisa et al. (2022)	Y	Y	Y	Y	Y	Y	N	Y	Y	Y	**9**
Mahlangu et al. (2022)	U	Y	Y	Y	Y	N	Y	Y	Y	Y	**8**
Marques et al. (2020)	U	Y	Y	Y	Y	U	N	Y	N	Y	**6**
McMahon et al. (2021)	U	Y	Y	Y	Y	N	U	Y	N	Y	**6**
Philyaw-Kotov et al. (2023)	U	Y	Y	Y	Y	Y	N	Y	Y	Y	**8**
Sall & Littleton, 2022	U	Y	Y	Y	Y	U	N	Y	Y	Y	**7**
Voth Schrag et al. (2024)	N	Y	Y	Y	Y	Y	N	N	Y	Y	**7**
Worthen and Wallace (2021)	Y	Y	Y	Y	Y	Y	N	Y	U	Y	**8**
Worthen and Wallace (2017)	U	Y	Y	Y	Y	U	N	Y	U	Y	**6**
Sum (total 21)	**8**	**21**	**21**	**21**	**20**	**11**	**7**	**19**	**15**	**21**	

JBI quality assessment and review instrument (QARI) (Joanna Briggs Institute, 2014). Y = yes; N = no; U = unclear.

Bolded numbers indicate summative qualitative assessment scores.

### Meta-Synthesis of Qualitative Research Findings

The meta-syntheses of the 21 included studies generated four synthesized topics: (1) dichotomous perceptions of SV initiatives, (2) the need for enhanced awareness, (3) modality matters, and (4) intended and unintended consequences. These synthesized topics were derived from 91 findings (e.g., themes) that were aggregated into 17 categories. The relationship of the synthesized topics, categories, and findings are displayed in Table 3.

**Table 3.** Relation of Synthesis Topics, Categories, and Findings.

Synthesis topics	Categories	Findings (*n* = themes)
Dichotomous perceptions of sexual violence initiatives	Conflicting views of programs and services (e.g., attention to SV is good, better reporting policies, available information in an ineffective format, triggering content)	17
	Lack of attention to diverse and intersectoral issues	15
	Content is not relatable	3
	A harm-reduction approach to learning is valued	1
	Need for comprehensive sexual health programs (healthy sexuality)	8
Need for enhanced awareness	Lack of acquired knowledge of programs and services	3
	Social media preferred to increase awareness of programming	2
	Marketing campaign was effective in perceived confidence in supporting a peer	1
	More specific information is needed in marketing campaigns	1
Modality matters	Constraining format	3
	Online training ineffective	5
	Learning was realistic and relatable	5
	Engaging and interactive programming is valued	4
	Peer-led programming is preferred	2
	Incorporate in existing campus activities (course or orientation)	5
Intended and unintended consequences	Enhanced outcomes with training (e.g., empowered to intervene, development of empathy and attitudes, support)	13
	Reporting (positive and negative experiences)	16

#### Dichotomous Perceptions of SV Initiatives

Students had mixed experiences regarding SV programs and services to prevent SV on campus. While many of the studies identified student perceptions of shortcomings in existing programs and services, some praised the university for bringing attention to the issue of SV and taking prevention efforts seriously. Participants who valued university initiatives indicated that learning about SV assisted them in recognizing and reporting sexual victimization and accessing resources (Voth Schrag et al., 2024). For example, an Asian heterosexual female participant who attended mandatory online sexual assault education stated, “*I’m glad they finally implemented a mandatory thing like this that covers* [sexual assault]. . . *I feel like this program will make students realize how serious this issue really is*” (Worthen & Wallace, 2017). Another participant who was a white undergraduate woman survivor of SV commended the university for their mandatory online sexual assault education program, “*As someone who has been a victim of past sexual misconduct, I was happy to see that attention was being brought to the issue*” (Worthen et al., 2021). Overall, students appreciated prevention programs that were both relatable and realistic (Christensen, 2014), thereby providing students with increased opportunities to intervene in potentially unsafe situations. One undergraduate Native American woman expressed the value she attributed to a university initiative by stating, “*I have a friend who thinks she might have been raped at a party because she was taken advantage of while drunk. The program helped me realize that she was assaulted*” (Worthen & Wallace, 2021). The outcomes that participants attributed to the learning demonstrate students’ perceived value of SV prevention programming on campus.

In contrast to the positive attributes of university SV initiatives, many participants expressed dissatisfaction with the programs and services. Some students disclosed that programming perpetuates rape myths such as “*all perpetrators are men*” and that perpetrators of sexual assault are “*strangers*.” These narrow perceptions of sexual assault did not reflect participant’s realities. In a study with women of color, one participant stated,

I don’t remember any space in terms of classroom or educational, institutionalized space where they talk about, oh, okay, what if it happens from someone you love, someone like a partner, domestic partner kind of thing. It was always talked about like, some random stranger is going to come for you. Not that that doesn’t happen, but I think it’s more often the case, these people who are in your life, like your close network kind of thing, how do you deal with that? (Karunaratne & Harris, 2022)

Given that participants’ experiences with SV often included a partner or acquaintance, the education was lacking in relatable content.

Most university communities include student representation from diverse groups inclusive of race, sexuality, gender, ability, and various backgrounds and experiences. However, many participants noted that the campus SV programming neglected the needs of the diverse student body. This led to decreased student comfort, and they perceived programming as less reflective of their identities and therefore ineffective. A male undergraduate student who attended an SV bystander program expressed the intersectional issues for black males, “*All the time, every time; black men can never be the victim, they are always the aggressor. They can be tied up, bound up within an inch of their life and somehow, they’re still wrong*” (Hammock et al., 2022). The perceptions identified by this participant reflect some of the challenges for black men to intervene when there is a risk of SV. They expressed that existing programming failed to address the negative current and historical perceptions of black men from society and the police, making intervention in SV risk scenarios personally dangerous. Overall, some diverse participants whose identities are not represented in campus programs and services may disengage or disconnect from the initiatives. Thus, students spoke of the need for diverse representation in campus services:

I think more counselors, a diverse set of counselors, is really important. I just don’t feel comfortable telling a white woman my problems. There is only white and Black—just get more diversity and have options for people so they can feel comfortable with someone who may look like them (undergraduate student). (Bloom et al., 2022)

In addition to diverse programs and services, participants also expressed the importance of campus-based initiatives that are realistic to student’s realities and are less absolute in their approach. For instance, some campus messaging included statements about the inability of someone to consent to sex while they were consuming alcohol. As the use of alcohol is a part of campus culture, students expressed that this is too “*black and white*,” and a harm-reduction approach is preferred. For example, a student with a disability disclosed their perception of alcohol and SV prevention programming on campus:

Everything felt way too absolute. I’m not necessarily in the agreement that, if someone has been drinking, they can’t consent to sex. I don’t think that that makes that much sense. I think it depends on the context. When a statement that’s just like, no, this is always wrong is made, if I don’t agree with it, it makes the whole thing less effective (Chugani et al., 2021)

Similarly, a student expressed the unrealistic messaging around consent, “The program effectively made men feel as though there is no way to ascertain consent without a notary present and a signed consent form” (Worthen et al., 2017). Participants called for a harm-reduction approach in campus SV initiatives that focus on safety in a realistic university context.

Participants also spoke about flipping the script with less focus on “*not getting sexually assaulted*” to a focus on “*how not to sexually assault someone.*” They also recommended developing comprehensive programming that explores healthy sexuality, and healthy relationships alongside issues of consent, bystander intervention, and a broad focus on sexual health and life skills, “*I think our programs spend so much time telling people what sex shouldn’t look like. I think it would help if we discussed what sex should look like. Sex should hit these three boxes: was it enjoyable, mutual, and consensual*” (woman, 21 years old) (Hubach et al., 2019). Finally, students disclosed that for content to be taken seriously, programming delivered by their peers (Greek life, athletes, etc.) would increase the relevance and “*break down barriers*” to receiving the message and accessing services.

#### Need for Enhanced Awareness

Despite an abundance of SV programs and services available on campuses, there was a general lack of awareness among students of existing initiatives to prevent SV and support sexual assault survivors. In an Australian study that evaluated an online SV prevention and response initiative, half of the students who participated in the study were unaware that a module existed on campus until they saw the poster advertising the research opportunity (Heard et al., 2024). Furthermore, once they became aware of the module, they found it difficult to access it on the university’s system. Similarly, in another study that evaluated US college students’ perceptions of mandatory reporting policies for sexual assault, only 10% of research participants could correctly identify and provide details of the existing policy (Holland et al., 2021) suggesting that there is a general lack of awareness of services on campus. These findings are consistent with findings from Marques et al. (2020) who indicated that 11/15 students were unaware of their university’s policies and procedures. Across various studies, students made statements such as “*the services are very limited*” or “*I don’t know*” or “*gosh, I’d have to google it*” when discussing their awareness of existing campus-based SV prevention programs and services. One female participant articulated the lack of awareness of their campus’s initiatives in SV, “*I actually don’t know anything about it. . .I don’t know* [how] *the procedure works or what they do, or the help number. . .I don’t know how they handle it. . .how they run things. . .I don’t know anything about your policies*” (Marques et al., 2020). Some students suggested that there is so much information given at once when they first attend college, that it is difficult to remember everything, while others simply stated that they genuinely did not know any campus-based SV initiatives existed.

Despite the overall lack of awareness, some participants appeared to know that programs and services existed on campus, but they lacked knowledge of the details of services. Incidentally, one female participant stated that their awareness is perceived rather than based on actual acquired knowledge, “*I don’t know about it. I know there is something in place just because I assume. . .but I don’t know the exact details*” (Holland et al., 2021). Students provided several suggestions for increasing awareness of campus-based programs and services including an increased social media presence, utilizing University learning services, and advertising through online outlets. A student (Charli) highlighted the existing outlets for raising awareness “a *bigger social media presence would be a good thing, because you know* [the University] *has such a big* [online] *presence and it has like a few thousand followers*” (Irvine-Collins et al., 2023). Participants in other studies also expressed the benefits of increasing awareness through social media, but they also promoted the use of courses (lectures), events, billboards, and posters to increase awareness of existing programs and services. In a study that explored student’s perceptions of a marketing campaign for the prevention of SV on campus, one focus group participant indicated that the campaign’s visibility and messaging, “*increased my likelihood to refer someone* [to campus support services]” (Irvine-Collins et al., 2023).

#### Modality Matters

Participants across the various studies had mixed feelings about the modality of delivery of SV prevention initiatives within the college/university. While some found the delivery of campus-based programming to be engaging, others described it as ineffective and constraining. Ultimately, despite mixed reviews, it was apparent that the modality of prevention initiatives was important to students and either enhanced or inhibited their level of engagement and learning.

In-person campus-based initiatives received mixed reviews from students. Some participants spoke of the constraining format of in-person programming, indicating that programming is often presented to large groups of students, thereby inhibiting participation and student’s ability to pose questions and clarify content. One South Asian female student described their dissatisfaction with the way the program was delivered, “*it was so many people, I didn’t feel safe enough to react to what was going on, to process or to ask questions*” (Karunaratne & Harris, 2022). Another student (male) felt that “a person is way more apt to pay attention when they have people presenting in front of them, rather than when they’re just sitting in front of a computer” (Philyaw-Kotov et al., 2023). Students also found in-person prevention programming to be superficial, and lacking attention to the identities and needs of a diverse student body, “*all of the* [actors in the videos] *were white. All of them were heterosexual. And* [they] *spoke in a way that assumed everyone was just like them*” (Erika, Indian woman) (Karunaratne & Harris, 2022). These barriers prevented students from fully engaging in the content as it did not reflect their realities. Other students noted that their failure to attend campus SV prevention programming was related to a sense of shame due to the social stigma of attending, and their priority of maintaining friendships or maintaining their masculine image. For example, Graham et al. (2021) conducted focus groups or individual interviews with students who attended an SV prevention workshop or those who had an opportunity to attend and did not. A female participant who did not attend the SV prevention initiative described her rationale for the decision, “*You kind of don’t wanna like, I dunno, kill your chances of making friends and getting relationships by going to this kind of stuff.*” As such, these in-person initiatives were deemed ineffective for some students who expressed that an open conversation that is less restrictive with a free-flowing dialogue and a small group format is preferred to the constraining nature of the program.

Though some students recounted the barriers of in-person programming, others indicated that participating in programming in person would make it easier to pay attention and focus on the content when compared to online. Participants indicated that program delivery that is co-constructed with students, engaging and realistic is preferred over the typical didactic initiatives that are common in campus settings. One student commented about the importance of engaging in programming, with a harm-reduction focus,

He’s very funny, he makes the conversation very relatable, and very casual. Not like, we’re telling you to never drink or never have sex, we’re just trying to tell you that if you’re gonna do it, we really want you to be safe about it. It’s a really nice perspective, cause obviously these things happen on a College campus. (Chugani et al., 2021)

Participants emphasized the value of peer-led initiatives that address holistic sexual health issues. Peer-led programming is believed to be more engaging and relevant and reflects students lived-experiences,

If other fraternity guys or sorority girls came to us as members of the Greek community and said, here’s what we should be doing. Or, if its athletes, people who are athletes at the same University. . .or graduate students who have been in our shoes. That would be a lot more effective. . .I feel comfortable to speak with you all because I know we’ve had similar experiences. That shared experience helps break down barriers on campus. (male, 18 years old) (Hubach et al., 2019)

In addition to in-person programming, many campus-based initiatives occurred through online formats and had the same mixed reviews from students. Some students indicated that the benefits of online programming are the accessibility, privacy, and convenience of the modality. However, other students described the modality of program delivery as not engaging or important, and the content as “*frustrating*,” “*dumb*,” and not worth their time. A white bisexual female student who participated in a 45-slide online campus SV prevention module noted, [the program] has “*valuable information presented in an ineffective medium*.” Some students disclosed that mandatory online programming is perceived as simply a “*check box*” or “*a necessary hoop to jump through*” before partaking in their studies. Therefore, when left to their own devices to complete the training online, many participants did not perceive the training as an immediate need and therefore did not take it seriously. While participants urged the institutions to “*deal with*” sexual assault on campus, one female student stated, “*sexual assault is a problem at all Colleges and I think Colleges should deal with that. Because I mean, we have online training now for everyone, but. . .is that really effective*” (Hubach et al., 2019)? Ultimately, students expressed that when they are attending university and leaving their family homes for the first time, they have other, more pressing issues on their minds that take priority for them. They suggested that campus programs should consider a “*life skills course*” during orientation week that includes diverse topics that are beneficial for starting a new chapter of their life but also includes issues of sexual assault,

I know there was some sort of course when we were a freshman that teaches you how to take notes and stuff and that doesn’t make any sense to me why there is a course on how to take notes and there isn’t one on, “your life is about to be completely different, you aren’t living at home anymore. This is a part of adulthood and part of adulthood is you having a voice and saying yes and no”; like a life skills course but have a large portion of it be sexual assault based (Charlie, graduate student). (Acquaviva et al., 2023)

#### Intended and Unintended Outcomes

Many participants who attended campus-based programs expressed positive outcomes from the training. This included an increased awareness of SV, which precipitated greater self-reflection of issues such as the language they use, or the assumptions they make about rape. For example, a female student (South Africa) who attended an SV risk reduction and self-defense program, expressed a change in their attitudes toward sexual assault following completion of the program, “*Before coming here* [workshop] *I thought that women are raped because of how they dress, now I know, that is not the case*” (Mahlangu et al., 2022). Another participant stated, “*I learned that I retain misinformation and attitudes about rape that I need to work to fix*” (McMahon et al., 2021). The programs helped students to self-evaluate their values, morals, and beliefs related to SV and fostered a sense of empathy. A participant who attended a theater-based bystander intervention program reflected on changes in their beliefs and views following completion of the program:

I try not to use the words “bitch” or “slut.” In terms of criticizing other people, I try not to think a girl is a slut because she’s dressed provocatively—it’s her right to dress however she wants, and it doesn’t necessarily mean she acts a certain way because of it. (McMahon et al., 2021)

In addition to changed attitudes, participants were empowered to act in situations of SV following the completion of the campus-based program. These actions included such things as intervening when they perceived someone was at risk of sexual assault, effectively using assertive communication regarding sex in intimate relationships, and utilizing physical and verbal resistance strategies when they were at risk of victimization. They became more vigilant to the risks of sexual assault and developed more confidence in creating boundaries, using physical self-defense, and becoming pro-social bystanders who intervene rather than ignore (Machisa et al., 2022). A male undergraduate student who completed bystander intervention programming on campus explained his intention to intervene when risks of SV were perceived, “*We have people in our house, people we don’t know and they’re creeps. I spend half my night distracting and all that. Just to protect girls, we do that. That’s what we do whenever we throw parties*” (Hammock et al., 2022). Another student who completed bystander intervention programming at a different College stated, “*I try to keep my eyes peeled for any suspicious behavior going on to ensure that rape does not occur.*” Not only did students feel empowered to intervene when others were potentially at risk of SV, but they also turned this learning inward and committed to participation in campus-based violence prevention initiatives to demonstrate their support and commitment to the importance of SV within the campus community.

Despite the positive gains following prevention programming, some student survivors who participated in the programming disclosed that the SV prevention programming was harmful. These students experienced flashbacks or intense memories of the assault and found the programming to be insensitive and triggering. One bisexual female survivor of SV stated,

I am a former sexual assault victim, . . .I began having post-traumatic stress disorder symptoms and flashbacks [during the training]. Clearly [the training] brought out some deep-seeded memories I was perfectly content in not visiting again. . . I felt. . .outrage at how useless and insensitive the training was to sexual assault victims. (Worthen et al., 2021)

In addition to programming that was harmful, many students identified mandatory sexual assault reporting through Title IX to be harmful in various ways. In several universities, sexual assault prevention services included mandatory reporting if a member of the campus community became aware that a sexual assault had occurred. Participants expressed that there was a sense of institutional betrayal when a mandatory report was made as they felt pressured to disclose when they were not ready to do so, and the institution failed to provide adequate support (Sall & Littleton, 2022). For example, a student stated, “*what happened with Title IX, I would say was just as bad, if not worse, than the traumatic event itself*” (Keres) (Lorenz et al., 2022). Students felt dismissed, blamed, and unsupported. Furthermore, they expressed that they experienced financial, emotional/psychological, interpersonal, and academic harm as a result of the mandatory reporting policies in effect in their university. A student expressed her dissatisfaction with the Title IX office following a sexual assault,

I spent the rest of my time in College fearing that I would run into my rapist, and they [Title IX] did literally nothing. They provided no protective measures or anything to make sure I could still continue my education. Basically, what I did was take a lot of online classes to make sure I wasn’t having to be on campus. . . I definitely didn’t get the full scope of my educational experience (Bia). (Lorenz et al., 2022)

These negative experiences led students to drop out of school, fail or fall behind in course work, lose scholarships or grants, lose their jobs or work fewer hours, and lose trust in the academic institution and the services that are alleged to offer to support to survivors. Students also disclosed that the timeline of the investigative process and the limited access to services resulted in increased harm rather than promoting recovery. Some students expressed that student-directed reporting is preferred.

## Discussion

This systematic review and meta-synthesis evaluated 21 qualitative studies of students’ experiences with CSA prevention initiatives. Various types of campus initiatives were included such as online mandatory education, bystander training, theater-based programs, and various resources and policies. This review is the first of its kind and provides insight and understanding regarding students’ experiences with CSA initiatives (see Table 4 for the Summary of Critical Findings).

**Table 4.** Critical Findings.

Summary of critical findings
• Students generally lacked an awareness of the prevention initiatives and services for SV on their campus
• Students valued that IHL is giving attention to the issue of SV but have widespread criticisms that many initiatives are not relatable and lack diversity
• The preferred modality of initiatives differed among students and a variety of CSA initiatives may be needed to appeal to students’ preferences
• While there are many benefits to CSA initiatives, there can be unintended consequences

The meta-synthesis identified that students value the attention given to SV by their institutions. However, our review highlighted that students have mixed reviews regarding campus-based SV initiatives. While students validated the need for colleges and universities to address and take the problem of SV seriously, they voiced displeasure with some aspects of programs. Students indicated that some initiatives promoted rape myth acceptance, had heteronormative assumptions regarding relationships, and lacked diversity regarding race, gender, and sexual orientation. Programs should consider the diversity of the student body when developing materials and consider how gender, culture, sexuality, ability, etc., are represented in CSA initiatives. For example, the incorporation of gender-neutral examples may minimize the criticism of prevention programs being “anti-male” (Messner, 2016), while also recognizing the statistics that females are overwhelmingly the victims of sexual assaults perpetrated by males. Furthermore, an intersectional approach to SV programming is required, which includes contexts relevant to diverse students such as LGBTQ+ and those with racialized identities.

Our findings highlight that there is not one single strategy to address the needs of all students, as the preferred modality (online, in-person, bystander, peer-led) differed among students. Diverse types of programs (e.g., peer-led, interactive, online, in-person) should be considered, which may appeal to different methods of teaching and learning so that students can engage with their preferred style of learning. The findings also emphasize that there is a need for enhanced awareness of campus initiatives. IHL should consider strategies such as campus-wide mass media and public service announcements to disseminate SV prevention information so that students are better aware of the programs and resources offered at their campus. Consideration for potential unintended outcomes of campus-based initiatives is also warranted. In particular, revictimization of survivors can occur when campus resources fail to address students’ needs for support (Holland & Cipriano, 2021), have reporting policies that do not align with students’ consent (Holland et al., 2021), are perceived as insensitive to student survivors (Worthen & Wallace, 2021), or do not address student safety (Lorenz et al., 2022).

The synthesized findings regarding students’ experiences with CSA initiatives are an important addition to the extant literature. The findings may be used to inform IHL regarding SV program development and/or modifications to ensure that initiatives meet the needs of students (see Table 5 for a summary of implications for practice, policy, and research). Our findings identified that students were not fully engaged in programming. To enhance student engagement, students want their realities represented in programs, utilizing multiple modalities for delivery. Moreover, having programs that address students’ needs may lead to better student engagement in CSA prevention programs, which may ultimately assist in reducing the incidence of sexual assault on campus. Thus, campus-based SV programs should have a broad scope to assist students; however, increased efforts must be made to ensure students know what is available to them. This is important given the evidence regarding effective preventative evidence-based interventions is still emerging (Bonar et al., 2022). A recent systematic review and meta-analysis on the effect of CSA prevention programs that included 80 studies found only a small effect (*g* = .15) on sexual assault attitudes and behaviors and no significant effect on sexual assault perpetration (Kettrey et al., 2023). In addition, the rates of SV across campuses have not changed much over the last 3 decades (Banyard et al., 2005; Rozee & Koss, 2001) despite IHL prevention programs and government initiatives. Thus, understanding the first-hand accounts of students who have participated in campus-based SV initiatives is needed to maximize engagement and optimize intended outcomes.

**Table 5.** Summary of Implications for Practice, Policy, and Research.

Implications for programming, policy, and research
• There is a need for enhanced student knowledge of programs and services
• Programs should consider multiple modalities for the delivery of SV initiatives to meet the diverse interests of the student body
• Programs should consider the diversity of the student body when developing materials and consider how gender, culture, sexuality, ability, etc., are represented in CSA initiatives
• Attention needs to be given to the unintended negative outcomes that may result from CSA programs or policies
• Future research on this topic should include a representation of diverse groups of students as participants
• Future studies should evaluate initiatives that include students’ preferences for CSA initiatives (e.g., comprehensive sexual health programs, incorporating SV content in existing campus activities (e.g., syllabus week, orientation)

### Strengths and Limitations

The strengths of our systematic review include adherence to the PRISMA guidelines for systematic reviews and the JBI methodology for conducting qualitative reviews. We also used a comprehensive search strategy to identify relevant students. All included studies were critically appraised as having a low or moderate risk of bias. As the purpose of our study was to conduct a synthesis of qualitative findings of students’ experiences with CSA initiatives, our findings solely reflect qualitative methodologies and do not include studies evaluating students’ experiences quantitatively. Similarly, as we only included peer-reviewed literature, there is a potential for publication bias as unpublished studies were not included. Additional limitations of the review are that the majority of included studies had samples that were predominately Caucasian, female, undergraduate students. While there were a few studies that solely focused on men or diverse groups of students they were limited. In addition, the studies were primarily conducted in the United States. These limitations may limit the generalizability of our findings to other countries and diverse groups of students.

## Conclusion

The systematic review and meta-synthesis of students’ experiences with CSA initiatives identified both satisfaction with CSA initiatives and areas for improvement. These findings are important given current government initiatives that IHL provide preventative SV resources and services to students on campus. Thus, the findings from this review may be used for program development or modification to enhance student engagement and ensure that the programs they are delivering are acceptable to the individuals for whom they were designed to help. Increasing student engagement may be one important strategy to increase program efficacy. Alternatively, initiatives that may result in harm to survivors require careful consideration.

## Supplemental Material

sj-docx-1-tva-10.1177_15248380241297332 – Supplemental material for Campus Sexual Assault: A Qualitative Review and Meta-Synthesis of Students’ Experiences of Campus Prevention Initiatives Supplemental material, sj-docx-1-tva-10.1177_15248380241297332 for Campus Sexual Assault: A Qualitative Review and Meta-Synthesis of Students’ Experiences of Campus Prevention Initiatives by Karen McQueen, Jodie Murphy-Oikonen and Jasmin Hamm in Trauma, Violence, & Abuse
