# Supplementary material for: Campus Sexual Assault: A Qualitative Review and Meta-Synthesis of Students’ Experiences of Campus Prevention Initiatives
Source: Trauma Violence Abuse. 2024 Nov 25;26(5):922–37. doi: 10.1177/15248380241297332 (PMC12569107; doi:10.1177/15248380241297332)
Supplement: sj-docx-1-tva-10.1177_15248380241297332 – Supplemental material for Campus Sexual Assault: A Qualitative Review and Meta-Synthesis of Students’ Experiences of Campus Prevention Initiatives [file sj-docx-1-tva-10.1177_15248380241297332.docx]

**Supplementary Table (S1)**

Search Terms

The full search strategy from the library scientist for PubMed included ((Campus sexual assault[Title/Abstract] OR sexual assault[Title/Abstract] OR rape[Title/Abstract] OR gender-based violence[Title/Abstract] OR sexual victimization[Title/Abstract]) AND (Prevention[Title/Abstract] OR intervention[Title/Abstract] OR education[Title/Abstract] OR bystander program[Title/Abstract] OR program[Title/Abstract] OR consent education[Title/Abstract] OR awareness[Title/Abstract])) AND (Student*[Title/Abstract] OR college student[Title/Abstract] OR undergraduate[Title/Abstract])
